# Supplementary material for: Characterization of an Agarophyton chilense Oleoresin Containing PPARγ Natural Ligands with Insulin-Sensitizing Effects in a C57Bl/6J Mouse Model of Diet-Induced Obesity and Antioxidant Activity in Caenorhabditis elegans
Source: Nutrients. 2021 May 27;13(6):1828. doi: 10.3390/nu13061828 (PMC8227508; doi:10.3390/nu13061828)
Supplement: Supplementary file 1 [file nutrients-13-01828-s001.zip › nutrients-1187539-supplementary.pdf]

Characterization of an *Agarophyton chilense* oleoresin containing PPARγ natural ligands with insulin-sensitizing effects in a C57BL/6J mouse model of diet-induced obesity and antioxidant activity *in Caenorhabditis elegans.*

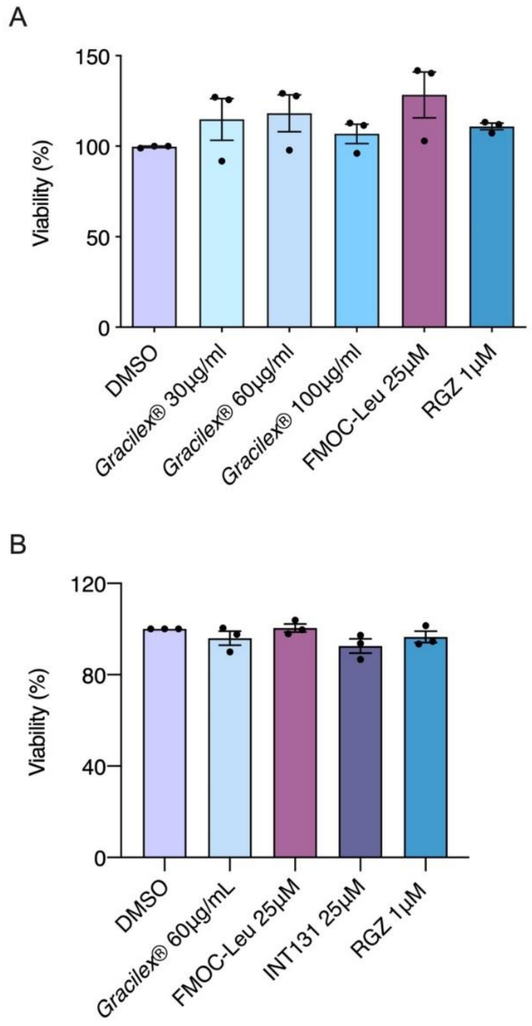

Supplementary Figure S1. Cell viability of PC12 and 3T3 cells after treatment with PPAR*γ* agonists and *A. chilense* oleoresin. (**A**) PC12 cells were cultured without or with FMOC-Leu, RGZ (1 µM) and *A. chilense oleoresin* or *Gracilex*® with three different concentration 30, 60, 90 µg/mL for 24 h. Then, the cells were incubated with MTT for 2 h at 37 ℃. MTT is used as an indicator of mitochondrial activity and cellular viability. The graph indicates viability relative to the control condition (treatment with vehicle, DMSO). No significant differences were found between treatments with the PPAR*γ* agonist and *Gracilex*® compared to the control (one-way ANOVA, Tukey's post hoc test). Values are expressed as the mean (n=3) ±SEM. (**B**) The 3T3-L1 preadipocytes were cultured without or with FMOC-Leu and INT131 at a concentration of 25 µM, rosiglitazone (RGZ, 1 µM) and *Gracilex*® (60 µg/mL), and at the end of the differentiation period, the cells were incubated with MTT for 2 h at 37 ℃. The graph indicates viability relative to the control condition (treatment with vehicle, DMSO). No significant differences were found between treatments with PPAR*γ* agonists and *Gracilex*® (one-way ANOVA, Tukey's post hoc test, n=3).

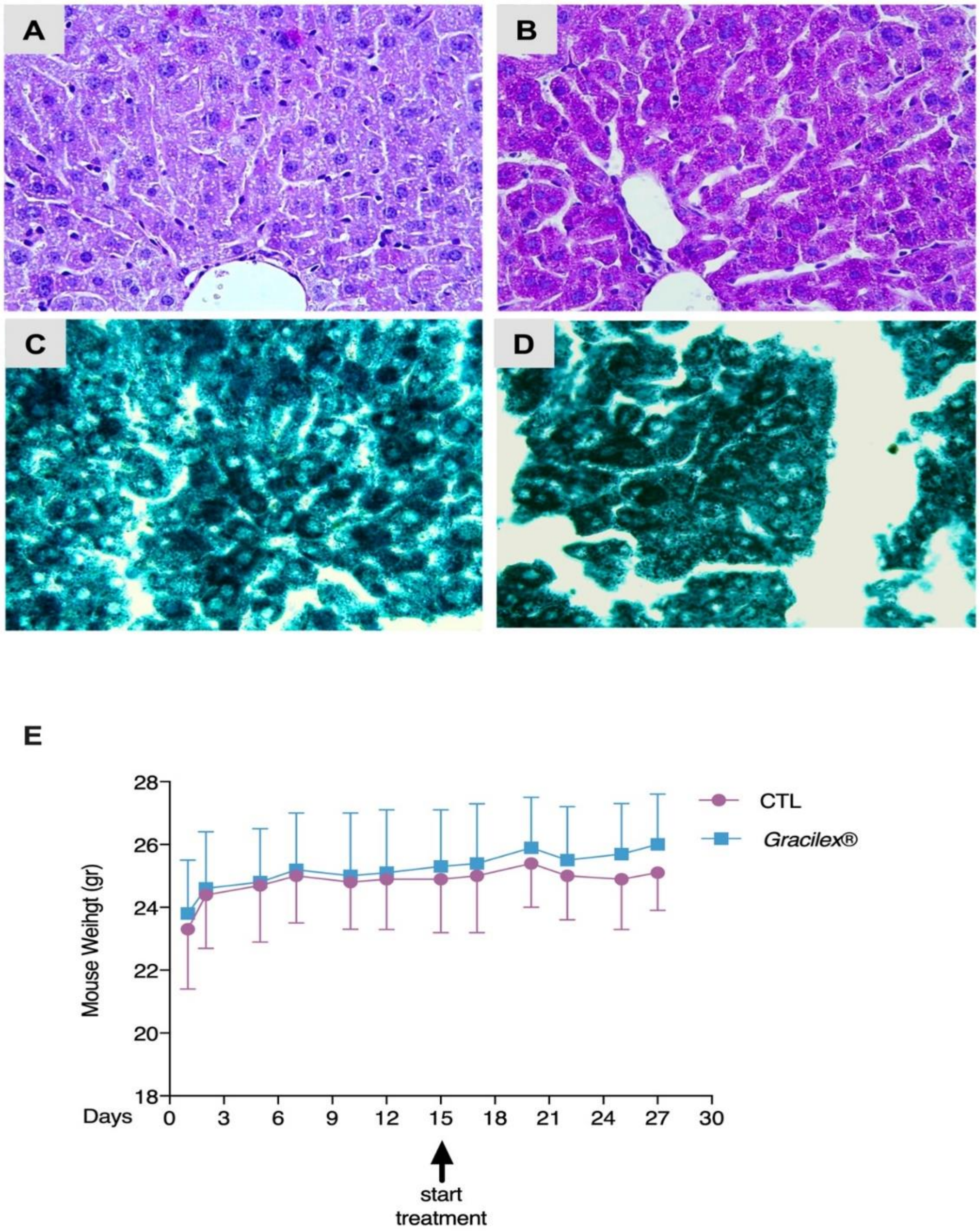

Supplementary Figure S2. Histological analysis of liver and weight measurements after chronic treatment with *A. chilense* oleoresin in mice fed a normal chow diet. Three-month-old C57BL/6J mice were treated daily for 15 days with 300 (mg/kg) of *Gracilex*® or corn oil (vehicle) extract. Light microphotograph of liver paraffin sections (400x). Hepatocytes were stained with periodic acidic-Schiff (PAS) staining to evaluate glycogen deposits (**A and B**) or with Sudan Black B staining for lipid accumulation (**C and D**). (**A**) and (**C**) are sections from control mice (vehicle). (**B**) and (**D**) are sections from control mice treated with *Gracilex*®. Mild to moderate glycogen storage was found in the group treated with *Gracilex*® compared to corn oil (vehicle). No difference was apparent in Sudan Black B staining between the groups. (**E**) Weight curves of normal chow-fed mice treated with *Gracilex*® for histological study. Three-month-old C57BL/6J mice (n=12 per group) were weighted 3 times per week, and after 15 days, daily treatment with *Gracilex*® 300 (mg/kg) or corn oil (vehicle) for an additional 15 days was started. Mice were weighted 3 times per week. The graph shows the average weight curve per group ±SEM.
